# Supplementary material for: Resolving the full spectrum of human genome variation using Linked-Reads
Source: Genome Res. 2019 Apr;29(4):635–45. doi: 10.1101/gr.234443.118 (PMC6442396; doi:10.1101/gr.234443.118)
Supplement: Supplemental Material [file supp_gr.234443.118_Supplemental_longranger-2.2.zip › longranger-2.2.tar.bz2!/lr2.2-public/lib/go/src/code.google.com/vitess/third_party/py/mock-1.0.1.tar.gz!/mock-1.0.1/html/magicmock.html]

Mocking Magic Methods — Mock 1.0.1 documentation


### Navigation

- index
- next |
- previous |
- Mock 1.0.1 documentation »

# Mocking Magic Methods¶

Mock supports mocking magic methods. This allows mock
objects to replace containers or other objects that implement Python
protocols.

Because magic methods are looked up differently from normal methods [1], this
support has been specially implemented. This means that only specific magic
methods are supported. The supported list includes *almost* all of them. If
there are any missing that you need please let us know!

You mock magic methods by setting the method you are interested in to a function
or a mock instance. If you are using a function then it *must* take self as
the first argument [2].

```
>>> def __str__(self):
...     return 'fooble'
...
>>> mock = Mock()
>>> mock.__str__ = __str__
>>> str(mock)
'fooble'

>>> mock = Mock()
>>> mock.__str__ = Mock()
>>> mock.__str__.return_value = 'fooble'
>>> str(mock)
'fooble'

>>> mock = Mock()
>>> mock.__iter__ = Mock(return_value=iter([]))
>>> list(mock)
[]
```

One use case for this is for mocking objects used as context managers in a
with statement:

```
>>> mock = Mock()
>>> mock.__enter__ = Mock(return_value='foo')
>>> mock.__exit__ = Mock(return_value=False)
>>> with mock as m:
...     assert m == 'foo'
...
>>> mock.__enter__.assert_called_with()
>>> mock.__exit__.assert_called_with(None, None, None)
```

Calls to magic methods do not appear in method\_calls, but they
are recorded in mock\_calls.

Note

If you use the spec keyword argument to create a mock then attempting to
set a magic method that isn’t in the spec will raise an AttributeError.

The full list of supported magic methods is:

- \_\_hash\_\_, \_\_sizeof\_\_, \_\_repr\_\_ and \_\_str\_\_
- \_\_dir\_\_, \_\_format\_\_ and \_\_subclasses\_\_
- \_\_floor\_\_, \_\_trunc\_\_ and \_\_ceil\_\_
- Comparisons: \_\_cmp\_\_, \_\_lt\_\_, \_\_gt\_\_, \_\_le\_\_, \_\_ge\_\_,
  \_\_eq\_\_ and \_\_ne\_\_
- Container methods: \_\_getitem\_\_, \_\_setitem\_\_, \_\_delitem\_\_,
  \_\_contains\_\_, \_\_len\_\_, \_\_iter\_\_, \_\_getslice\_\_,
  \_\_setslice\_\_, \_\_reversed\_\_ and \_\_missing\_\_
- Context manager: \_\_enter\_\_ and \_\_exit\_\_
- Unary numeric methods: \_\_neg\_\_, \_\_pos\_\_ and \_\_invert\_\_
- The numeric methods (including right hand and in-place variants):
  \_\_add\_\_, \_\_sub\_\_, \_\_mul\_\_, \_\_div\_\_,
  \_\_floordiv\_\_, \_\_mod\_\_, \_\_divmod\_\_, \_\_lshift\_\_,
  \_\_rshift\_\_, \_\_and\_\_, \_\_xor\_\_, \_\_or\_\_, and \_\_pow\_\_
- Numeric conversion methods: \_\_complex\_\_, \_\_int\_\_, \_\_float\_\_,
  \_\_index\_\_ and \_\_coerce\_\_
- Descriptor methods: \_\_get\_\_, \_\_set\_\_ and \_\_delete\_\_
- Pickling: \_\_reduce\_\_, \_\_reduce\_ex\_\_, \_\_getinitargs\_\_,
  \_\_getnewargs\_\_, \_\_getstate\_\_ and \_\_setstate\_\_

The following methods are supported in Python 2 but don’t exist in Python 3:

- \_\_unicode\_\_, \_\_long\_\_, \_\_oct\_\_, \_\_hex\_\_ and \_\_nonzero\_\_
- \_\_truediv\_\_ and \_\_rtruediv\_\_

The following methods are supported in Python 3 but don’t exist in Python 2:

- \_\_bool\_\_ and \_\_next\_\_

The following methods exist but are *not* supported as they are either in use by
mock, can’t be set dynamically, or can cause problems:

- \_\_getattr\_\_, \_\_setattr\_\_, \_\_init\_\_ and \_\_new\_\_
- \_\_prepare\_\_, \_\_instancecheck\_\_, \_\_subclasscheck\_\_, \_\_del\_\_

# Magic Mock¶

There are two MagicMock variants: MagicMock and NonCallableMagicMock.

*class* MagicMock(*\*args*, *\*\*kw*)¶
:   MagicMock is a subclass of Mock with default implementations
    of most of the magic methods. You can use MagicMock without having to
    configure the magic methods yourself.

    The constructor parameters have the same meaning as for Mock.

    If you use the spec or spec\_set arguments then *only* magic methods
    that exist in the spec will be created.

*class* NonCallableMagicMock(*\*args*, *\*\*kw*)¶
:   A non-callable version of MagicMock.

    The constructor parameters have the same meaning as for
    MagicMock, with the exception of return\_value and
    side\_effect which have no meaning on a non-callable mock.

The magic methods are setup with MagicMock objects, so you can configure them
and use them in the usual way:

```
>>> mock = MagicMock()
>>> mock[3] = 'fish'
>>> mock.__setitem__.assert_called_with(3, 'fish')
>>> mock.__getitem__.return_value = 'result'
>>> mock[2]
'result'
```

By default many of the protocol methods are required to return objects of a
specific type. These methods are preconfigured with a default return value, so
that they can be used without you having to do anything if you aren’t interested
in the return value. You can still *set* the return value manually if you want
to change the default.

Methods and their defaults:

- \_\_lt\_\_: NotImplemented
- \_\_gt\_\_: NotImplemented
- \_\_le\_\_: NotImplemented
- \_\_ge\_\_: NotImplemented
- \_\_int\_\_ : 1
- \_\_contains\_\_ : False
- \_\_len\_\_ : 1
- \_\_iter\_\_ : iter([])
- \_\_exit\_\_ : False
- \_\_complex\_\_ : 1j
- \_\_float\_\_ : 1.0
- \_\_bool\_\_ : True
- \_\_nonzero\_\_ : True
- \_\_oct\_\_ : ‘1’
- \_\_hex\_\_ : ‘0x1’
- \_\_long\_\_ : long(1)
- \_\_index\_\_ : 1
- \_\_hash\_\_ : default hash for the mock
- \_\_str\_\_ : default str for the mock
- \_\_unicode\_\_ : default unicode for the mock
- \_\_sizeof\_\_: default sizeof for the mock

For example:

```
>>> mock = MagicMock()
>>> int(mock)
1
>>> len(mock)
0
>>> hex(mock)
'0x1'
>>> list(mock)
[]
>>> object() in mock
False
```

The two equality method, \_\_eq\_\_ and \_\_ne\_\_, are special (changed in
0.7.2). They do the default equality comparison on identity, using a side
effect, unless you change their return value to return something else:

```
>>> MagicMock() == 3
False
>>> MagicMock() != 3
True
>>> mock = MagicMock()
>>> mock.__eq__.return_value = True
>>> mock == 3
True
```

In 0.8 the \_\_iter\_\_ also gained special handling implemented with a
side effect. The return value of MagicMock.\_\_iter\_\_ can be any iterable
object and isn’t required to be an iterator:

```
>>> mock = MagicMock()
>>> mock.__iter__.return_value = ['a', 'b', 'c']
>>> list(mock)
['a', 'b', 'c']
>>> list(mock)
['a', 'b', 'c']
```

If the return value *is* an iterator, then iterating over it once will consume
it and subsequent iterations will result in an empty list:

```
>>> mock.__iter__.return_value = iter(['a', 'b', 'c'])
>>> list(mock)
['a', 'b', 'c']
>>> list(mock)
[]
```

MagicMock has all of the supported magic methods configured except for some
of the obscure and obsolete ones. You can still set these up if you want.

Magic methods that are supported but not setup by default in MagicMock are:

- \_\_cmp\_\_
- \_\_getslice\_\_ and \_\_setslice\_\_
- \_\_coerce\_\_
- \_\_subclasses\_\_
- \_\_dir\_\_
- \_\_format\_\_
- \_\_get\_\_, \_\_set\_\_ and \_\_delete\_\_
- \_\_reversed\_\_ and \_\_missing\_\_
- \_\_reduce\_\_, \_\_reduce\_ex\_\_, \_\_getinitargs\_\_, \_\_getnewargs\_\_,
  \_\_getstate\_\_ and \_\_setstate\_\_
- \_\_getformat\_\_ and \_\_setformat\_\_

---

|  |  |
| --- | --- |
| [1] | Magic methods *should* be looked up on the class rather than the instance. Different versions of Python are inconsistent about applying this rule. The supported protocol methods should work with all supported versions of Python. |

|  |  |
| --- | --- |
| [2] | The function is basically hooked up to the class, but each Mock instance is kept isolated from the others. |

### Table Of Contents

- Mocking Magic Methods
- Magic Mock

#### Previous topic

Sentinel

#### Next topic

Getting Started with Mock

### This Page

- Show Source

### Quick search


Enter search terms or a module, class or function name.

### Navigation

- index
- next |
- previous |
- Mock 1.0.1 documentation »

© Copyright 2007-2012, Michael Foord & the mock team.
Last updated on Nov 05, 2012.
Created using Sphinx 1.1.3.
